# Efficacy of repetitive transcranial magnetic stimulation at different sites for peripheral facial paralysis: a prospective cohort study

**DOI:** 10.3389/fneur.2023.1285659

**Published:** 2023-11-03

**Authors:** Zicai Liu, Xin Wen, Yuchun Shao, Zihao Wan, Bangliang Liu, Risheng Wang, Huiyu Liu

**Affiliations:** ^1^Department of Rehabilitation Medicine, Shaoguan First People’s Hospital, Shaoguan, China; ^2^Department of Rehabilitation Medicine, YueBei People’s Hospital, Shaoguan, China; ^3^College of Physical Education and Health, Guangzhou University of Chinese Medicine, Guangzhou, China; ^4^The First Affiliated Hospital of Nanchang University, Nanchang, China

**Keywords:** repeated transcranial magnetic stimulation, cohort study, peripheral facial paralysis, peripheral facial neuritis, repetitive transcranial magnetic stimulation

## Abstract

**Background:**

There are very few studies on transcranial magnetic stimulation (TMS) therapy for facial paralysis and no studies comparing the efficacy of central and peripheral TMS in the treatment of peripheral facial paralysis (PFP).

**Purpose:**

To observe the therapeutic effect and security of central and peripheral repetitive transcranial magnetic stimulation (rTMS) on PFP.

**Methods:**

Patients with unilateral onset of peripheral facial paralysis within 1 month were prospectively recruited, 97 patients with PFP were divided into the peripheral group, central group, and control group. The control group was given common treatment (drug therapy and acupuncture), and the peripheral and central groups received rTMS in addition to conventional treatment. After 2 weeks of treatment, the House-Brackmann (HB) grading scale, Sunnybrook facial grading system (SFGS), and modified Portmann scale (MPS) were used to evaluate the facial muscle function of patients in the three groups.

**Result:**

After 2 weeks of rTMS treatment, the HBGS/SFGS/MPS scores of the three groups were significantly better than before (*p* < 0.05), and the mean change values of HBGS, SFGS, and MPS scores were significantly higher in participants in Peripheral Group (*p* < 0.001; *p* < 0.001; *p* = 0.003; respectively) and Central Group (*p* = 0.004; *p* = 0.003; *p* = 0.009; respectively) than in Control Group. But the mean change values of HBGS, SFGS, and MPS scores showed no significant differences in participants in the Peripheral Group than in the Central Group (*p* = 0.254; *p* = 0.139; *p* = 0.736; respectively) after 2 weeks of treatment (*p* > 0.05).

**Conclusion:**

Our study shows that rTMS can be a safe and effective adjuvant therapy for patients with PFP. Preliminary studies have shown that both peripheral and central stimulation can effectively improve facial nerve function, but there is no significant difference in the efficacy of the two sites.

## 1. Introduction

Peripheral facial paralysis (PFP) is a loss of function of the facial nerve-innervated tissues, which may be partial or total, presenting with facial muscle dyskinesia, often resulting in facial asymmetry persisting for many weeks or months (1). The exact cause of the disease is unknown (2, 3), it is reported to occur in about 20–30 cases per 100,000 people (4). Several treatments are usually used, including antivirals, Vitamin B drugs, steroids, surgery, physiotherapy, acupuncture treatment, and others (5). Although 70 percent of patients are cured within a year, 30 percent still suffer sequelae of varying degrees (6). This is still a high percentage, so finding new ways to improve outcomes is necessary.

Transcranial magnetic stimulation (TMS) is another promising technology that is attracting significant attention. As a noninvasive brain stimulation technique, repeated transcranial magnetic stimulation (rTMS) has been reported to accelerate recovery from peripheral facial neuritis (7). Few studies have focused on the application of rTMS intervention in facial paralysis. Most of the previous studies have focused on the clinical value of TMS in the electrophysiological diagnosis of PFP (8, 9). Through the preliminary experiment and clinical observation, we believe that rTMS has a certain rationality and value in the treatment of peripheral facial neuritis.

The purpose of this study was to determine the efficacy of repeated transcranial magnetic stimulation (rTMS) for peripheral facial neuritis and to compare the curative effects of peripheral stimulation and central stimulation.

## 2. Method

### 2.1. Study design

This study was a prospective, 3-arm, no-randomized controlled trial. Patients with PFP who participated in the study were either outpatients or inpatients between 2021 and June 2022 at Yuebei People’s Hospital. Depending on the type of intervention, all patients were assigned to three parallel groups: peripheral, central, and control groups. All patients provided written informed consent. The clinical trials by Yuebei People’s Hospital Medical Ethics Committee approval (approval number of KY - 2021-075), this research project has been registered in the Chinese clinical trial registry (http://www.chictr.org.cn/) (registration number: ChiCTR2100053550).

### 2.2. Participants

We prospectively enrolled patients aged 18–75 years who were diagnosed with peripheral facial neuritis at the Yuebei People’s Hospital. The initial screening process includes tests and evaluations, including electromyography or electroneurogram examination, in addition, imaging and laboratory tests are performed to rule out brain damage or other causes of facial paralysis secondary to facial paralysis. All the patients we included were required to have the first onset and unilateral facial paralysis, and the course of the disease was required to be within 1 month, and the grading of House-Brackmann scale (10) was ≥3 level, willing to cooperate with the researchers for treatment and evaluation and sign the informed consent form. Exclusion criteria are facial paralysis due to stroke, encephalitis and Lyme disease and various tumors, intracranial metal foreign bodies, epilepsy, pregnancy, and other transcranial magnetic contraindications in patients, patients with other serious illnesses that were unstable or in the acute phase were also excluded, any other conditions that the investigator thought would affect the experiment may be considered excluded from the study.

### 2.3. Interventions

**Figure 1:**
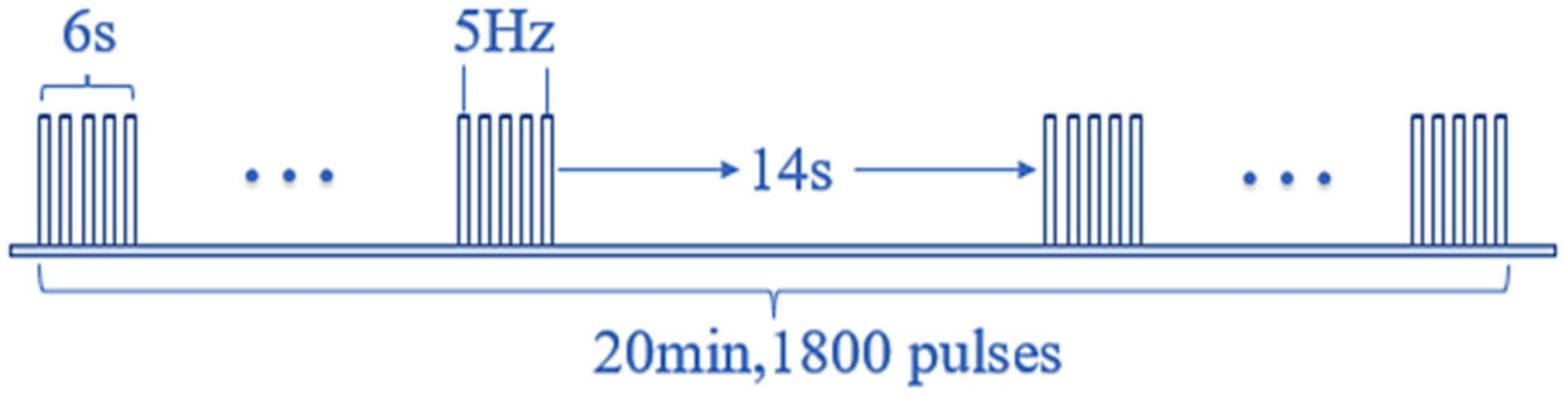
Schematic diagram of the pulse of rTMS.

All three groups received conventional antiviral drugs and neurotrophic drugs, as well as the same acupuncture treatment, the medication regimen and adherence to medications did not change throughout the study. Both peripheral and central groups received repetitive transcranial magnetic stimulation (Jiangxi Brain Regulation Technology Development limited-liability company, NTK-TMS-II transcranial magnetic stimulation instrument), and the stimulus coil was a figure of 8 coil (size: 104∗196∗16 mm). The stimulation program has been used to treat facial paralysis (11). The peripheral and central groups received the same rTMS regimen, Magnetic Pulse Parameter Specifications: Stimulation frequency is 5 Hz, stimulation time of a single pulse string is 6 s, the interval between pulse strings is 14 s, so a pulse cycle is the 20 s, and the number of input pulses for each pulse cycle is 30; each time the treatment is repeated for 60 cycles, the treatment time of each transcranial magnetic stimulation is 1,200 s (20 min), and the total number of output pulses is 1,800 pulses. All patients in the central and peripheral groups received 10 sessions of rTMS over 2 weeks, for a total of 18,000 pulses, a maximum of one rTMS per day. The schematic diagram of the pulse is shown in Figure 1.

**Figure 2:**
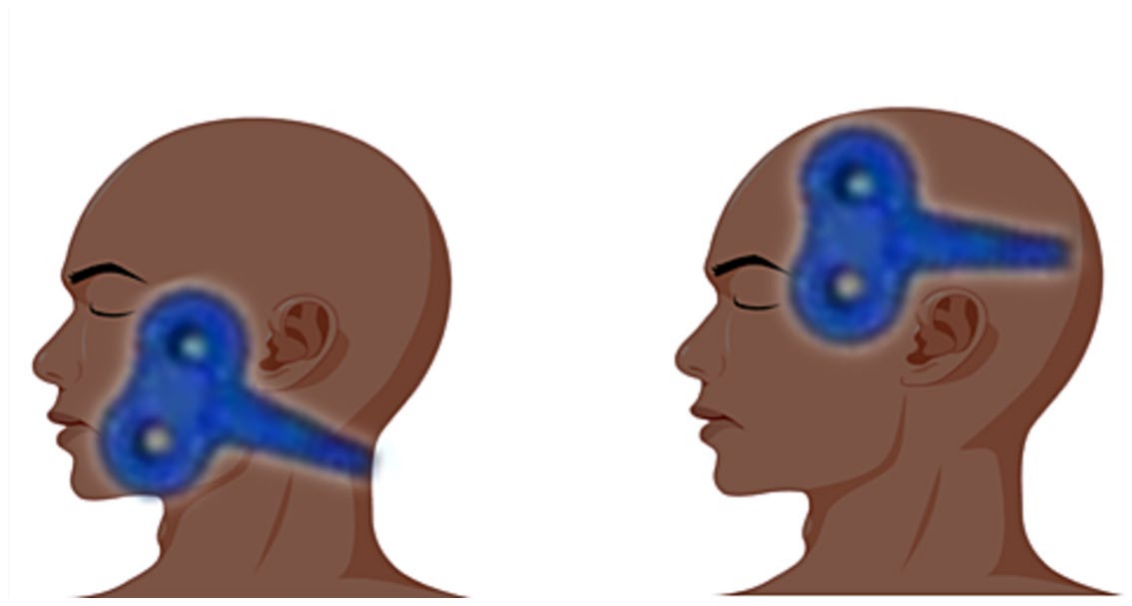
The image on the left shows peripheral stimulation and the image on the right shows central stimulation.

The stimulation site of the central stimulation group was the M1 area (7), Positioning caps were designed following the EEG 10–20 electrode placement system. The patient wears a cap that fits his/her head circumference, and the coil is placed in the corresponding position of the “face” in the cap pattern for stimulation (between the areas of F7/F3/T3/C3, the facial motor cortex area). This method is now more widely used in China, the positioning cap is clearly labeled, easy to use, and can be a good solution to the problem of rapid positioning during clinical treatment; in the peripheral group, the stimulation site was on the face of the injured side, located between the mandibular notch and the lower margin of the zygomatic arch (11), the coil stimulation site is shown in Figure 2.

### 2.4. Determination of the resting motor threshold

Resting motor thresholds (RMT) were measured before intervention in all patients receiving rTMS. The patient was asked to sit quietly and remain relaxed, approximately 3–6 CM from the apex is the center of the “8” coil, and a disposable electrode with 2 diameters of 1 cm was symmetrically fixed to the nasal muscle, recording nasal muscle complex muscle action potential (CMAP) after transcranial magnetic stimulation (TMS) (9, 11). RMT is determined by visual inspection of minor muscle contractions. We had a similar situation: in most subjects, it was not sufficient to contract the targeted muscle (nasal muscle) alone (12), and additional contraction of the tongue or masticatory muscle or both were required to obtain a response. To determine the intensity required for a maximal response, the healthy side was first stimulated (9). We referenced the RMT measured on the first day for only one measurement, and subsequent stimuli were referenced to this intensity. Perhaps due to our technique, only a small number of patients could measure the RMT of the nasolabial muscles, and the majority of patients could only test the FDI, so in practice both were present, and we understand that this may lead to some bias, but in most cases, the compound muscle action potentials of the first dorsal interosseus (FDI) muscles were recorded as well, FDI helps to localize the coil over the motor cortex and select the appropriate TMS intensity for each patient (13), this method widely used in stroke patients. The motor-evoked potential (MEP) of the FDI muscle was used as a reference to determine the strength only when the MEP of the nasal muscle is not fired or difficult to fire. During measuring FDI, the coil plane was attached to the scalp section and kept parallel, the coil handle was all oriented to the occipital side, and the coil was at a 45° Angle to the sagittal line of the subject. RMT was taken as the minimum stimulus intensity that caused a slight contraction of the target muscle in 5 out of 10 stimuli (14). Generally, the stimulation intensity of patients during treatment is between 80 and 120% RMT. However, in this study, we adjusted the percentage of RMT according to the patient’s tolerance.

### 2.5. Outcome assessment

We used three different assessments of facial nerve function as outcome measures. It consisted of the Sunnybrook Facial Grading System (SFGS) (15), the House-Brackmann Grading Scale (HBGS) (10), and the Modified Portmann Scale (MPS) (16). The SFGS score ranges from 0 to 100, with higher scores indicating better facial nerve function, and measures static symmetry, voluntary symmetry, and synchronized facial movements. HBGS are graded from 1 to 6, with higher grades being more severe, and are assessed for facial symmetry at rest, forehead, eye, and mouth motor function during movement, and overall associated movement (11, 17). The MPS assesses the completion of six actions (raising eyebrows, closing eyes, widening nostrils, showing teeth, pursing mouth, bulging cheeks) on a scale of 0–20 across four dimensions, with higher scores indicating better facial function (7, 11, 18).

In addition, we recorded any adverse events that occurred during the study. The evaluators were not aware of patient groupings and interventions (blindness).

All patients were individually assessed by two professionals, and the average value of the two assessors was subsequently taken as a record, the evaluator did not know to which group the patient was assigned (blind). The intervention the evaluation and data analysis were all made up of different researchers, unaware of each other’s specific work, and finally pooled by another researcher.

### 2.6. Statistical analysis

The data of this study were analyzed by SPSS 26.0 software. Using the Shapiro–Wilk test, we determined that the data distribution is normal. Continuous variables were measured using the mean or median of interquartile spacing; the Chi-square test was used for dichotomous variables such as gender in baseline data. One-way analysis of variance or nonparametric test was used according to whether the data were in line with normal distribution and homogeneity of variance. Paired *T*-test or rank sum test was used to compare the same data before and after the trial intervention. *p* < 0.05 was considered statistically significant.

## 3. Results

**Figure 3:**
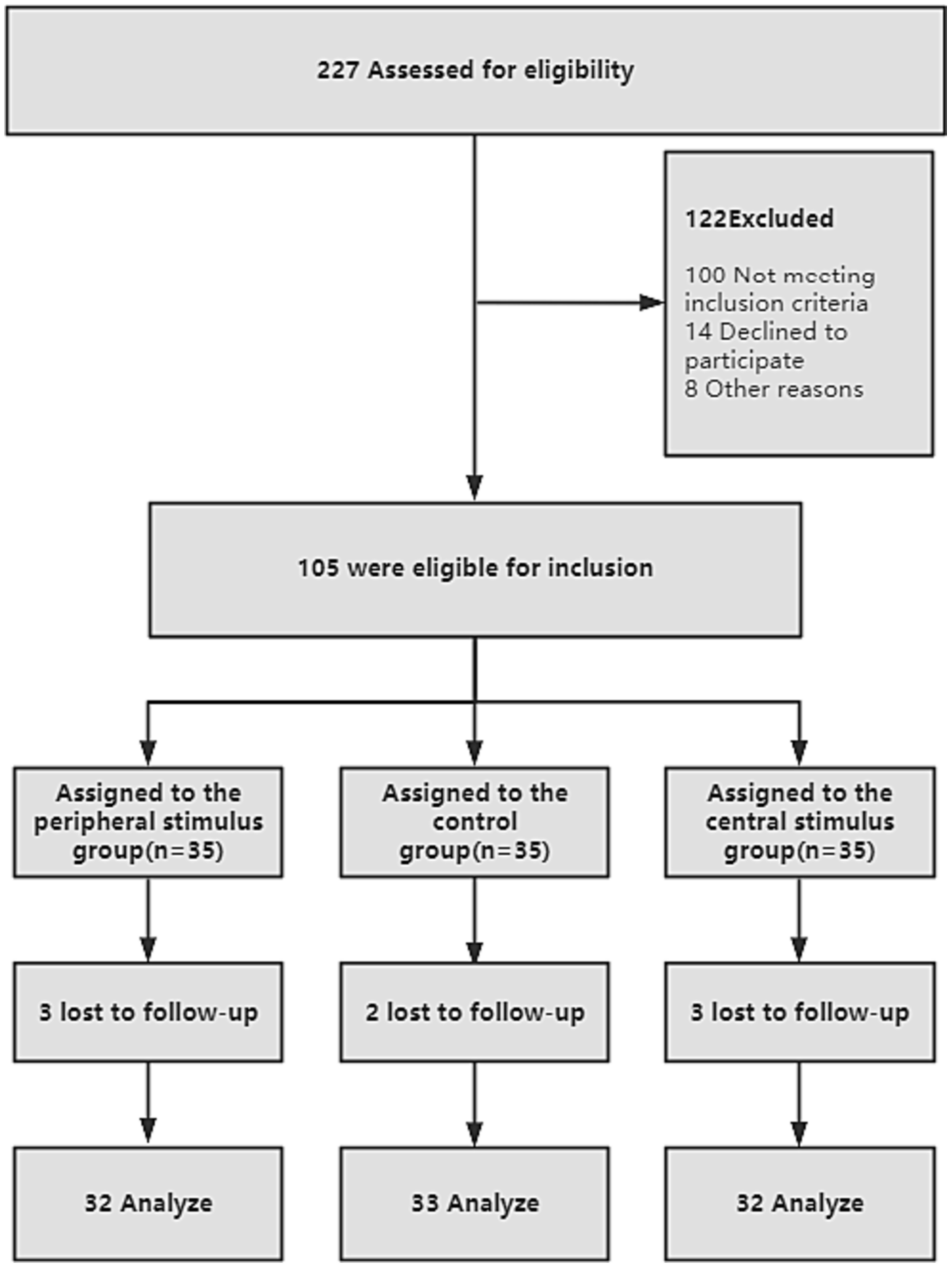
Participant flow diagram.

We recruited 227 patients with facial paralysis, of whom 122 did not meet the criteria and were excluded, and 105 met the inclusion criteria. An average of 35 patients in each group were assigned to central stimulation, peripheral stimulation, and control groups. In the early stages of the trial, eight people withdrew from the trial for reasons of force majeure (Lack of time, hospital transfer, etc), and eventually, 97 completed all trials and were included in the analysis (Figure 3).

The baseline characteristics of the three groups are shown in Table 1. There were no significant differences in disease duration, age, sex ratio, hypertension, diabetes, and other characteristics among the three groups (*p* > 0.05), this suggests that the three groups are comparable.

**Table 1:** Baseline characteristics of the three groups.

Characteristics	Peripheral group (*n* = 32)	Central group (*n* = 32)	Control group (*n* = 33)	*p*
Sex (F/M)	19/13	14/18	16/17	0.439
Age (years)	45.6 ± 12.1	51.1 ± 14.6	49.6 ± 16.1	0.297
Facial paralysis side, (left/right)	16/16	15/17	14/19	0.827
The course of disease (day)	6.9 ± 5.3	7.0 ± 6.6	5.0 ± 3.9	0.250
Hypertension (yes/no)	7/25	9/23	7/26	0.772
Diabetes (yes/no)	5/27	5/27	4/29	0.897
Resting motor thresholds (RMT)	40.1 ± 10.3	40.3 ± 9.4	/	0.930
HBGS	4.7 ± 0.6	4.7 ± 0.5	4.5 ± 0.7	0.089
SFGS	23.3 ± 12.9	25.8 ± 11.4	31.8 ± 17.7	0.053
MPS	5.2 ± 2.2	5.3 ± 1.7	5.9 ± 2.9	0.509

HBGS, House-Brackmann grading scale; SFGS, Sunnybrook facial grading system; MPS, modified Portmann scale; IQR, interquartile range; SD, standard deviation.

The results showed that there was no significant difference in HBGS, SFGS, and MPS before treatment among the three groups (see Table 1). But After 2 weeks of transcranial magnetic therapy, there were significant improvements after treatment compared with before treatment, and the differences were statistically significant (*p* < 0.05, see Table 2).

**Table 2:** Comparison of efficacy before and after treatment among three groups.

Group	HBGS		SFGS		MPS	
	Pretreatment	Postreatment	Pretreatment	Postreatment	Pretreatment	Postreatment
Peripheral	4.7 ± 0.6	2.3 ± 0.9*	23.3 ± 12.9	74.5 ± 21.5*	5.2 ± 2.2	14.8 ± 4.0*
Central	4.7 ± 0.5	2.5 ± 0.8*	25.8 ± 11.4	71.0 ± 18.5*	5.3 ± 1.7	14.6 ± 3.1*
Control	4.5 ± 0.7	2.8 ± 0.8*	31.8 ± 17. 7	64.4 ± 23.1*	5.9 ± 2.9	12.9 ± 4.2*

HBGS, House-Brackmann grading scale; SFGS, Sunnybrook facial grading system; MPS, modified Portmann scale; *p < 0.05 (Pretreatment vs. Postreatment).

Using the mean change before and after intervention as an indicator, we made a horizontal comparison of the three outcome indicators of the three groups and constructed a histogram. The mean change values of the HBGS score, SFGS score, and MPS score between pre-treatment and post-treatment of the three groups (*F* = 8.847, *p* < 0.001; *F* = 10.690, *p* < 0.001; *F* = 5.410, *p* = 0.006; respectively) were statistically significant. After 10 times of rTMS treatment, the mean change values of HBGS, SFGS, and MPS scores were significantly higher in participants in the Peripheral Group (*p* < 0.001; *p* < 0.001; *p* = 0.003; respectively) and Central Group (*p* = 0.004; *p* = 0.003; *p* = 0.009; respectively) than in Control Group (Table 3). However, the mean change values of HBGS, SFGS, and MPS scores showed no significant differences in participants in the Peripheral Group than in the Central Group (*p* = 0.254; *p* = 0.139; *p* = 0.736; respectively) after 2 weeks of treatment (Table 3).

**Table 3:** Multiple comparisons of changes in the assessment of facial function among the three intervention groups.

Change in mean	Peripheral group vs. central group (*p* value)	Peripheral group vs. control group (*p* value)	Central group vs. control group (*p* value)
HBGS	0.254	<0.001	0.004
MPS	0.736	0.003	0.009
SFGS	0.139	<0.001	0.003

HBGS, House-Brackmann grading scale; SFGS, Sunnybrook facial grading system; MPS, modified Portmann scale.

None of the patients involved in this study experienced serious adverse events; two patients in the Peripheral group had a mild toothache, and no patients reported toothache when the placement of the stimulation coil was appropriately adjusted (11), This situation occurs when the treatment is carried out once, mostly due to the coils of transcranial magnetism directly stimulate the teeth resulting in discomfort, and further retention will induce toothache, after adjusting the position of the coils, it will not directly stimulate the teeth, but stimulate the nerves and muscles around the face, so it is necessary for the treatment staff to adjust the coils in time. One patient had a mild headache in the central group, and the headache disappeared after reducing the intensity of TMS. No seizures, vomiting, or other adverse reactions occurred, and no other discomfort and withdrawal from clinical studies, indicating a better safety profile and high compliance with rTMS for PFP.

## 4. Discussion

This was a prospective cohort study of 97 patients with peripheral facial neuritis to compare the efficacy of central vs. peripheral rTMS. The results showed that after 2 weeks of treatment, the HBGS/SFGS/MPS scores of the three groups were significantly improved compared with those before treatment. The mean changes of HBGS/SFGS/MPS of the peripheral group and the central group were significantly higher than those of the control group, but there was no significant difference in the mean change before and after treatment between the central group and the peripheral group. This suggests that both central and peripheral stimulation had the same efficacy, and the efficacy of rTMS combined with conventional treatment was greater than that of conventional treatment alone. To the best of our knowledge, this is the first study to compare the efficacy of central rTMS and peripheral rTMS against PFP. Previous studies have only demonstrated the effectiveness of central stimulation (7) or peripheral stimulation (11) alone, and relevant studies in this field are lacking at present.

PFP is caused by a malfunction in the facial nerve that prevents facial muscles from being controlled (19), the clinical manifestations include difficulty frowning on the affected side, inability to close the eyes, air leakage from bulging cheeks, weakness in closing the lips, etc. Transcranial magnetic stimulation was used for this disease initially for electrophysiological diagnosis, Meyer et al. used TMS to measure facial nerve block in patients with facial paralysis (13), and Nowak et al. found TMS seems capable of localizing the site of the lesion within the Fallopian channel (9). Rimpiläinen et al. showed that TMS-induced facial motor responses predicted a good prognosis in early Peripheral facial neuritis (20). With the development of TMS and people learning more about facial paralysis, TMS is used as an intervention for the recovery of facial paralysis. Lan Shaoyong et al. used TMS combined with conventional treatment for PFP and found that the HBGS and the complete recovery rate of the observation group were significantly better than that of the control group (21). Liang and Qiang suggest TMS helped improve the clinical symptoms and EMG metrics in patients with facial paralysis and improved the clinical efficacy and patient prognosis, along with good safety (22). Our previous study using rTMS on the affected face for peripheral stimulation found that peripheral rTMS could also significantly improve facial nerve function in patients with Bell facial paralysis (11).

Although we found that both central and peripheral stimulation improved PFP, the mechanism may be different. For peripheral TMS stimulation, the mechanism may be to improve the nutritional supply to the injured facial-related nerves (23) and promote blood flow (24), in addition, when the TMS coil is placed on the affected side of the face of a PFP patient, it produces muscle contractive vibrations. This mechanical vibration (25) and electrical stimulation stimulate the muscle spindles of the facial muscles and strengthen nerve control over the muscles. This peripheral electrical stimulation also activates sensory nerves, the trigeminal nerve plays an important role. Cheney et al. (26) and Martin and Helsper (27) have observed the possibility of *de novo* neuralization of the trigeminal nerve in paralyzed facial muscles, electrically induced orthodromic excitation of the trigeminal nerve fibers may promote the generation of terminal motor nerve twigs (28). In addition, another important explanation is that TMS relies on a magnetic field to generate induced current, and the magnetic field of the coil will touch the affected face, so peripheral TMS also has the effect of magnetic therapy on the affected face tissue. Magnetic therapy improves circulation, reduces inflammation, and reduces pain (29, 30). Different from the mechanism of peripheral TMS stimulation, the effective mechanism of central TMS stimulation may be the use of high-frequency (excitatory) TMS elicitation to guide the activation of the brain cortex representative area of facial movement, which is helpful to the remodeling of the facial muscle motor function, and the nerve impulse excited will also project to the facial nervous system, to accelerate the recovery of facial nerve function (31). There have been multiple studies showing that in patients with peripheral facial paralysis, the representative area of the cerebral cortex related to facial muscle decreases (32), the adjacent area (such as the forearm muscle cortex representative area) increases, and the functional remodeling of facial motor cerebral cortex representative area is closely related to the prognosis of patients with peripheral facial paralysis (20, 33, 34). Functional magnetic resonance imaging (fMRI) studies by Hu et al. suggested that cortical reorganization plays an important role in the recovery of Bell’s facial paralysis (35). Facial nerve dysfunction has a destructive effect on the activity of sensorimotor areas, and the increased intensity of sensorimotor areas ipsilateral to the facial nerve injury in the middle stage of facial nerve dysfunction suggests that interhemispheric reorganization may be involved. Behavioral or brain stimulation techniques in this phase of treatment can be used to alter the reorganization of sensorimotor areas in facial functional rehabilitation, monitor treatment effects, and improve therapeutic interventions during rehabilitation (36).

For a look at the future, we have summarized some of the unresolved things. First, there is uncertainty regarding the optimal target of TMS for PFP, and our preliminary findings of no significant difference between central and peripheral stimulation are not absolute. Yang et al. used TMS to stimulate the outlet mastoid of the facial nerve and found that the percentage of R1 extraction rate and the percentage of CMAP amplitude decline of the facial nerve in the intervention group were significantly higher than those in the control group, suggesting that rTMS has a good clinical effect on the treatment of early PFP (37), more research will be needed to compare the efficacy of these different sites. Secondly, it is necessary to optimize the optimal treatment parameters of TMS. The current studies generally use stimulation frequencies of 5 HZ and 50 HZ (iTBS) (37), but there is no study to compare the efficacy difference of TMS with different frequencies. Finally, whether TMS is specific for PFP patients at different stages and how effective it is in the sequelae stage needs to be further explored. A point that needs to be discussed directly is this: if peripheral stimulation (and perhaps even the use of more convenient and less expensive electrical stimulators) is equally effective, is there a need for TMS, and is there a need to design a study comparing the effects of peripheral electrical stimulation with those of magnetic stimulation? We believe that this is a question that deserves in-depth research, and our answer is YES, we look forward to future studies that can compare the effects of peripheral electrical stimulation with magnetic stimulation or have a relevant systematic review to prove it.

This study has the following limitations: First of all, we did not use electrophysiological indicators such as electromyograms and only used HBGS, SFGS, and MPS as outcome indicators, which was somewhat subjective. Second, we did not calculate the sample size, and it was not randomly assigned, which may weaken the evidence quality of the study.

In conclusion, our study shows that rTMS can be used as a safe and effective adjuvant therapy for patients with PFP. Preliminary studies have shown that both peripheral and central stimulation can effectively improve facial nerve function, but there is no significant difference in the efficacy of the two sites.

## Data availability statement

The original contributions presented in the study are included in the article/supplementary material, further inquiries can be directed to the corresponding authors.

## Ethics statement

The studies involving humans were approved by Ethics Committee of Yuebei People’s Hospital. The studies were conducted in accordance with the local legislation and institutional requirements. The participants provided their written informed consent to participate in this study. Written informed consent was obtained from the individual(s) for the publication of any potentially identifiable images or data included in this article.

## Author contributions

ZL: Conceptualization, Investigation, Software, Writing – original draft, Writing – review & editing. XW: Methodology, Writing – original draft, Writing – review & editing. YS: Data curation, Formal analysis, Methodology, Project administration, Writing – original draft. ZW: Data curation, Formal analysis, Project administration, Writing – original draft. BL: Formal analysis, Project administration, Supervision, Validation, Writing – original draft. RW: Investigation, Methodology, Project administration, Writing – original draft. HL: Conceptualization, Funding acquisition, Investigation, Resources, Supervision, Validation, Visualization, Writing – original draft.
